# Investigation of floristic similarities between Taiwan and terrestrial ecoregions in Asia using GBIF data

**DOI:** 10.1186/s40529-017-0171-0

**Published:** 2017-03-27

**Authors:** Chi-Cheng Liao, Chih-Hui Chen

**Affiliations:** 1 Department of Life Science, Chinese Culture University, 55, Hwa-Kang Road, Yang-Ming-Shan, Taipei, 11114 Taiwan, ROC; 2 Endemic Species Research Institute, Nantou County, Taiwan, ROC

**Keywords:** Angiosperm, Asia, Continental island, Floristic similarity, Geographical distribution, Taiwan, Terrestrial ecoregion

## Abstract

**Background:**

Floristic compositions of non-endemic plants of continental islands were related to the neighboring continents because non-endemic plant species had historically migrated to continental islands from source areas. This study attempts to identify source areas of a continental island by means of floristic analysis and to assess possible migration routes on the basis of geographical distribution ranges of plants. Large quantities of angiosperm data records were downloaded from the Global Biodiversity Information Facility (GBIF). Similarity index and cluster analysis were used to identify the floristic similarities among 22 geographical localities of Taiwan (GLTs) and 34 terrestrial ecoregions in Asia. Geographical distribution ranges of non-endemic angiosperm species in Taiwan (NEASTs) were evaluated to mirror the possible migration routes from different source areas to Taiwan.

**Results:**

There are 3275 angiosperm species in Taiwan derived from the dataset of GBIF. Among them, 847 are endemic and 2428 are NEASTs. Geographical distribution ranges of the 2428 NEASTs were categorized into 7 distribution groups. They were widely distribution from equator to Siberia (27 species), tropical ecoregions (345 species), tropical and subtropical ecoregions (663 species), tropical to temperate ecoregions (591 species), subtropical ecoregions (265 species), subtropical to temperate ecoregions (387 species), and temperate ecoregions (150 species). Results of similarity indices and cluster analysis demonstrated that high floristic similarities were observed among GLTs at lowland and southern Taiwan and tropical and subtropical ecoregions in Asia. GLTs at high mountains were assumed to have floristic similarity with temperate ecoregions in Asia, whereas the assumption was not supported by our analysis. It is partly because of that angiosperms with tropical and subtropical distributions extend their ranges from low to high elevations in Taiwan.

**Conclusions:**

Subtropical ecoregions at southern China and tropical ecoregions at Indochina were more important than temperate ecoregions on playing source areas of NEASTs. Geographical distribution ranges of NEASTs implied that most of the NEASTs were probably migrated from topical or subtropical ecoregions of Asian continent to Taiwan.

**Electronic supplementary material:**

The online version of this article (doi:10.1186/s40529-017-0171-0) contains supplementary material, which is available to authorized users.

## Background

Oceanic islands possess disproportionately high plant species richness and numbers of endemic taxa (Bramwell and Caujapé-Castells 2011; Brooks et al. 2002; Carlquist 1967; Chen and He 2009; Cowie and Holland 2006; Kier et al. 2009; Kreft et al. 2008; Krupnick et al. 2009; Malcolm et al. 2006; Waldren et al. 1995). On the contrary, continental islands possess relatively lower proportion of endemic taxa and floras of continental islands are closely related to floras of neighboring continents (Bramwell and Caujapé-Castells 2011; Fernández-Palacios et al. 2011; Heaney 1991; Hsu and Wolf 2009). In East Asia, plant species richness of continental islands are closely related to the Eurasian continents and neighboring regions (Chiang and Schaal 2006; Hiramatsu et al. 2001; Nakamura et al. 2009; Ota 1998; Setoguchi et al. 2008). The extent to which non-endemic species of continental islands are related to the neighboring regions remains elusive.

Taiwan locates at the eastern border of Eurasian continent and was formed by the collision between Luzon Arc and Eurasian continent during 2–3 million years ago (Chen and Liu 1996; Hsieh et al. 2006; Liew and Hsieh 2000; Teng 1990, 2007; Voris 2000; Wei 2002; Zeng 1993). Land bridge connections between Taiwan and Eurasian continent (Hsieh et al. 2006; Liew and Hsieh 2000) allowed immigration of plants from neighboring regions to Taiwan (Chiang and Schaal 2006). The proportion of endemic vascular plant species of Taiwan is 26.1%, while more than 73% of vascular plants in Taiwan are not endemic (Hsieh 2002). Non-endemic plant species might have migrated to Taiwan from the neighboring regions, or the source area. Nonetheless, that where had been the source areas of plants in Taiwan is an unanswered question.

Since few decades ago, tens of botanists had interested on the source areas of angiosperm species in Taiwan. Several studies had addressed on the phylogenetic relationships of plants among Taiwan and neighboring regions, including Japan, Ryukyu archipelago and China, to evaluate the immigration of plants from neighboring regions to Taiwan (Chen et al. 2009; Chiang and Schaal 2006; Huang et al. 2002; Kokubugata et al. 2011; Wei et al. 2010). Some studies had focused on the floristic compositions to explore the species richness of Taiwan and floristic relationships among Taiwan and neighboring regions (Chao et al. 2010; Feroz and Hagihara 2008; Hsu and Wolf 2009; Liao et al. 2013; Tang et al. 2013). All the phylogenetic and floristic studies attempted to answer two questions: where were the source areas of flora of Taiwan and how many plants had migrated from tropical, subtropical or temperate regions to Taiwan, respectively. The questions remain unanswered because continuous vegetation coverage extends from Malay Peninsula northward to the Arctic of Siberia and floristic compositions of vegetations change from tropical to temperate regions (Fang et al. 2002; Ni 2003; Ohsawa 1993, 2006; Olson et al. 2001). The question which vegetation types or which regions are the important source areas of insular non-endemic plant species in Taiwan have never been investigated.

Taiwan is characterized by the massif of a central mountain system with the highest peak of ca. 4000 m above sea level (ASL) and Tropic of Cancer crosses the southern part of the island. The northern and southern part of Taiwan belongs to the subtropical and tropical climate zones, respectively. Climates change from southern to northern areas and from low to high elevations in Taiwan (Chen 1957). Diverse climatic conditions in Taiwan are influencing the plant distributions and differentiation of floristic compositions within the island (Chiou et al. 2010; Su 1984, 1985). Six geographical areas was divided in Taiwan in terms of climatic environments, plant distributions and floristic compositions (Su 1984, 1985). Floristic differentiation among six geographical areas in Taiwan leads to two assumptions. The first is that different floristic composition of geographical areas in Taiwan had presumably related to different source areas in Asia. The second is that there had been probably several migration routes for plants to migrate from different source areas to Taiwan.

This study attempts to identify the source areas of non-endemic angiosperm species in Taiwan (NEAST). Floristic similarities between Taiwan and neighboring terrestrial ecoregions in Asia were analyzed to identify the possible source areas. Geographical distribution ranges of the NEASTs in Asia were explored to mirror the possible migration routes of angiosperms from different source areas to Taiwan.

## Methods

### Data collection and preparation

Large quantities of angiosperm data records were utilized to analyze floristic relationships and to evaluate geographical distribution ranges of plants. The angiosperm data records of all the countries in East and South Asia were downloaded from the Global Biodiversity Information Facility (GBIF, http://www.GBIF.org). More than 4.5 millions data records were collected. The data records of angiosperms were mapped by Diva-GIS (Hijmans et al. 2001). Data records with incorrect values of latitude and longitude or without scientific names were dropped out from the analysis.

### Angiosperm list of Taiwan

Angiosperm list of Taiwan is the first and the most important dataset of this study. There are two available angiosperm lists of Taiwan from two different data sources. The first list (List I) was derived from the Flora of Taiwan, including 3420 angiosperm species (Huang 1996). The second list (List II) was derived from the plant data records downloaded from the GBIF and the number of data records was 560,000. There are more than 8000 scientific names in the List II. The numbers of angiosperm species are different between List I and List II because some synonyms are included in the two lists. The corrections and revisions of the scientific names from GBIF and incorporation of the two lists were performed.

Most of the taxa have the same scientific name in both List I and List II, whereas some taxa have different scientific names between List I and List II. If so, the scientific names from GBIF (List II) were recognized as accepted names in this study, while that from Flora of Taiwan (List I) synonyms. The scientific names of the List I and List II were checked on the website of GBIF to identify whether the names were accepted names or synonyms. Synonyms were substituted by accepted names in GBIF and accepted names were reserved in the two lists. The accepted names both occurred in the List I and List II were collected and incorporated into List III. As a result, a total of 3275 angiosperm species were confirmed in the List III (see Additional file 1: Appendix S1).

### Geographical localities of Taiwan

**Fig. 1:**
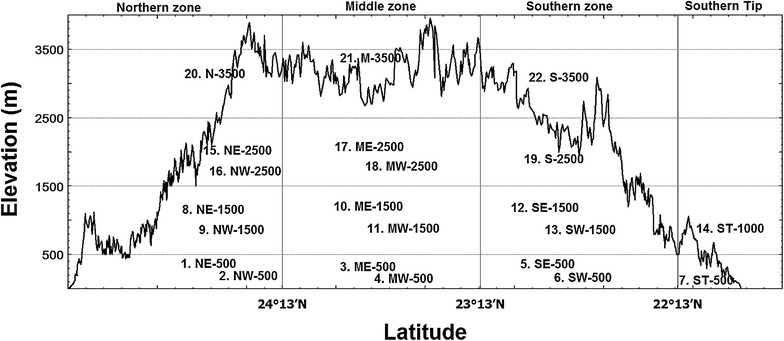
The numbers and acronyms of 22 geographical localities of Taiwan (GLTs). The *solid curve* represents the profile of the island. Taiwan was divided into four latitude zones, northern zone (N), central zone (C), southern zone (S), and southern tip (ST). The N, C and S latitude zones were divided into eastern (E) and western (W) slopes of central mountain system. Seven geographical areas were obtained, they were NE, NW, CE, CW, SE, SW and ST. The seven geographical areas were then divided into several geographical localities according to elevation. The GLTs were numbered from north to south and from low to high elevations

The spatial distributions of plants in Taiwan are strongly affected by climate, elevation, topography, and monsoon winds (Chao et al. 2010; Chen et al. 1997; Chiou et al. 2010; Hsieh et al. 1997, 1998; Li 2014; Li et al. 2013; Mabry et al. 1998; Su 1984, 1985; Zhang et al. 2010). Taiwan had been divided into six geographical areas in terms of diverse climatic environments and floristic compositions (Su 1984, 1985). Based on the six geographical areas of Su (1984), we divided Taiwan into several geographical localities (Fig. 1). First, Taiwan was divided into four latitude zones. The southern tip (ST) is at south of 22°13′ latitude and included Hengchun peninsula and Lanyu. The latitude of southern zone (S) is between 22°13′ and 23°13′ latitude, the central zone (C) is between 23°13′ and 24°13′ latitude, and the northern zone (N) is at north of 24°13′ latitude. The N, C and S latitude zones were further divided into eastern (E) and western slopes (W). The border between E and W of the three latitude zones is the mountain ridge of central mountain system, which runs from north to south of the island. Seven geographical areas, namely northeast (NE), northwest (NW), central east (CE), central west (CW), southeast (SE), southwest (SW), and ST, were obtained in our study. Next, each geographical area was divided into several geographical localities. The NE, NW, CE and CW were divided into 3 geographical localities below 2500 m ASL and the altitudinal ranges of geographical localities are 0–500 m ASL (lowland and foothill), 500–1500 m ASL (below the cloud zone), and 1500–2500 m ASL (cloud zone). Above 2500 m ASL (high elevation above the cloud zone), only one geographical locality was obtained for each of N and C latitude zones. Division of the S latitude zone is different because the highest elevation is relatively lower. Each of SE and SW was divided into 2 geographical localities below 1500 m ASL and the altitudinal ranges of geographical localities are 0–500 and 500–1500 m ASL. From 1500 to 3500 m ASL, 2 geographical localities, 1500–2500 and 2500–3500 m ASL, were obtained for the S latitude zone. The ST zone was divided into 2 geographical localities because elevation of the highest peak is lower than 1000 m ASL. Based on this method, Taiwan was divided into 22 geographical localities. The 560,000 data records of angiosperms from GBIF were divided into 22 data files of 22 geographical localities of Taiwan (GLTs) according to the latitude, longitude and elevations of data records. The 22 data files were utilized to generate 22 angiosperm lists of 22 GLTs.

### Terrestrial ecoregions in Asia

**Fig. 2:**
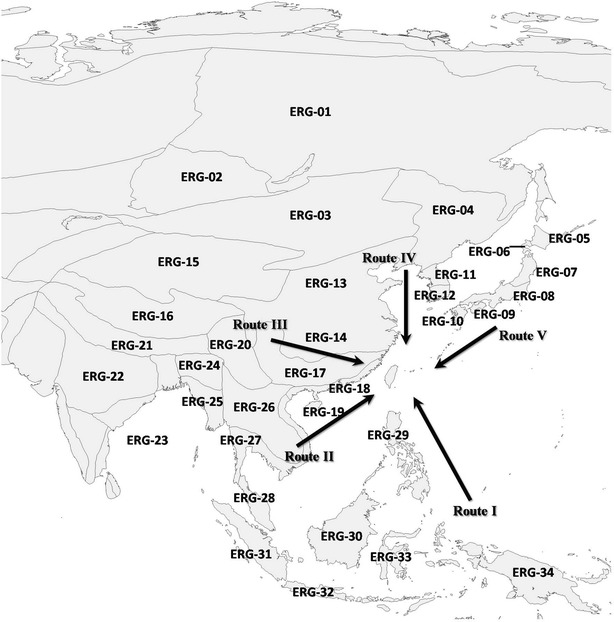
Map shows the locations of 34 ecoregions in this study. The acronyms of ecoregions are the same as in Table 1. Floristic relationships among Taiwan and 34 ecoregions were analyzed in this research. The *arrows* show the five hypothetical migration routes of angiosperms from tropical island (Route I), tropical continent (Route II), subtropical regions (Route III), temperate regions (Route IV) and temperate islands (Route V) to Taiwan (see “Discussion”)

The source areas in relation to the angiosperm species richness of Taiwan range from tropical islands at equator to subarctic Siberia and extend westward to western slope of Qinghai–Tibet Plateau. The source areas might have included continental Asia, India, Indochina, tropical islands and temperate islands in East Asia. However, the aforementioned regions are inappropriate to be used as analyzing units of floristic relationships. Latitude ranges and area sizes of the aforementioned regions or countries are wide enough to encompass distinct vegetation types. Instead of the aforementioned regions, terrestrial ecoregions proposed by World Wild Fund (WWF) (Olson et al. 2001) were utilized for the floristic analysis (Fig. 2). There are 867 terrestrial ecoregions, classified into 14 different biomes on earth. The descriptions of the terrestrial ecoregions can be checked on the website (http://wwf.panda.org/about_our_earth/ecoregions/ecoregion_list/) and the map of the delineations of the ecoregions was downloaded from the internet (http://www.worldwildlife.org/publications/terrestrial-ecoregions-of-the-world). It is because of that the ecoregions more accurately reflect the complex distribution of the Earth’s natural communities (Olson et al. 2001).

According to the published division of WWF, 38 terrestrial ecoregions in Asia are included in our study (Olson et al. 2001). However, local studies are not consistent with the division of the ecoregions. The northern and central China was categorized as one ecoregion in WWF, while some local references divided northern and central China into two floristic regions (Fang et al. 2002; Qiu et al. 2011). The Qinling Mountains–Huai River line (at ca. 34°N) demarcates the northern and central China (Qiu et al. 2011). Therefore, two ecoregions were divided by the Qinlin Mountains-Huai River line in this study; the northern China was categorized as temperate ecoregion (No. 13) and the central China subtropical ecoregions (No. 14). In addition, there are 6 ecoregions in Indian subcontinent according to WWF. The 6 ecoregions in Indian subcontinent was incorporated into one ecoregion because of two reasons. Floristic relationships between Taiwan and India were supposed to be lower and the number of plant data records at Indian subcontinent is relatively lower. The ecoregion in Indian subcontinent was numbered as 22. Finally, 34 ecoregions were obtained (Table 1; Fig. 2). Moreover, the 34 ecoregions were classified into four groups in terms of locations and climate characteristics. The four groups are cold-winter desert, temperate ecoregions, subtropical ecoregions, and tropical ecoregions (Table 1).

**Table 1:** The names and abbreviations of biomes and 34 terrestrial ecoregions in Asia

Acronym of ecoregions	Name of biome	Name of ecoregions	Number of data record^a^	Number of scientific name^b^
Group of cold winter deserts				
ERG-15	Cold-winter desert	Takla-Makan-Gobi Desert	12,062	2744
ERG-16	Cold-winter desert	Tibetan	38,004	2371
Group of temperate ecoregions				
ERG-02	Mixed mountain system	Altai highlands	2355	162
ERG-03	Temperate grasslands	Mongolian-Manchurian Steppe	2071	600
ERG-01	Temperate needle-leaf forests/woodlands	East Siberian Taiga	1343	467
ERG-04	Temperate broad-leaf forests	Manchu-Japanese mixed forests	3180	909
ERG-05	Temperate broad-leaf forests	Manchu-Japanese mixed forests	5575	988
ERG-06	Temperate broad-leaf forests	Oriental deciduous forests	442	238
ERG-07	Temperate broad-leaf forests	Oriental deciduous forests	33,562	2066
ERG-11	Temperate broad-leaf forests	Oriental deciduous forests	3618	1085
ERG-13	Temperate broad-leaf forests	Oriental deciduous forests	13,876	2959
Group of subtropical ecoregions				
ERG-08	Subtropical/temperate rain forests/woodlands	Japanese evergreen forest	287,306	4433
ERG-09	Subtropical/temperate rain forests/woodlands	Japanese evergreen forest	22,180	1857
ERG-10	Subtropical/temperate rain forests/woodlands	Japanese evergreen forest	1684	611
ERG-12	Subtropical/temperate rain forests/woodlands	Japanese evergreen forest	600,788	4519
ERG-14^c^	Temperate broad-leaf forests	Oriental deciduous forests	139,697	7862
ERG-17	Subtropical/temperate rain forests/woodlands	Chinese subtropical forests	194,854	11,696
ERG-20	Mixed mountain system	Szechwan highlands	186,933	12,271
ERG-21	Mixed mountain system	Himalayan highlands	55,145	6413
Group of tropical ecoregions				
ERG-18	Tropical humid forests	South Chinese rainforests	9396	2586
ERG-19	Tropical humid forests	South Chinese rainforests	5770	1876
ERG-23	Tropical humid forests	Bengalian rainforest	2230	412
ERG-25	Tropical humid forests	Burman rainforest	992	439
ERG-27	Tropical humid forests	Indochinese rainforests	196,220	5577
ERG-28	Tropical humid forests	Malayan rainforests	14,573	3339
ERG-22	Tropical dry forest/woodlands	India subcontinent	28,662	3112
ERG-24	Tropical dry forests	Burma monsoon forests	2075	573
ERG-26	Tropical dry forests/woodlands	Thailandian monsoon forest	33,293	5996
ERG-29	Mixed island systems	Philippines	33,825	3390
ERG-30	Mixed island systems	Borneo	90,332	6813
ERG-31	Mixed island systems	Sumantra	11,763	2341
ERG-32	Mixed island systems	Java	7431	1503
ERG-33	Mixed island systems	Sulawesi	20,724	2391
ERG-34	Mixed island systems	Papuan	226,960	9502

Locations of the ecoregions are showed on the map in Fig. 1. ERG is the abbreviation of ecoregion. The 34 ecoregions were categorized into 4 groups. Numbers of data records were downloaded from the Global Biodiversity Information Facility (GBIF) and numbers of scientific names were derived from the data records

^a^Number of data record: data records were downloaded from the GBIF and data records of an ecoregion were extracted from different countries and incorporated into one ecoregion

^b^Number of scientific name: the number of scientific names derived from the data records of GBIF. The number is not the real species richness because many synonyms were in the lists

^c^ERG-14 is categorized into subtropical group according to local references and different from the categorization of WWF

### Angiosperm lists of terrestrial ecoregions

The angiosperm data records of GBIF were downloaded by the countries in Asia. The countries in continental Asia include Russia, Mongolia, Korea, China, India, countries in Indochina, and Malaysia. The temperate islands belong to Japan and tropical islands include Philippine, Sumatra, Java, Borneo, Sulawesi, and New Guinea. More than 4.5 millions of angiosperm data records were downloaded from the GBIF. The boundaries of countries in Asia are different from that of ecoregions. In order to make angiosperm lists of ecoregions, angiosperm data records within an ecoregion were extracted from different countries. The angiosperm records extracted from different countries were manipulated by ArcMap software (ESRI, Redlands, USA). The extracted data records were incorporated into an ecoregion. The incorporated data records of ecoregions were mapped, checked and revised by Diva-GIS (Hijmans et al. 2001) and were utilized to generate angiosperm lists of ecoregions. Numbers of data records and numbers of angiosperm lists of ecoregions are listed in Table 1.

The angiosperm lists of ecoregions were compared with that of List III (angiosperm list of Taiwan). Non-endemic angiosperm species in Taiwan (NEAST) were evaluated their geographical distribution ranges in Asia. The NEASTs of each ecoregion were collected to generate angiosperm lists and, eventually, there are 34 lists of NEASTs of ecoregions in this study.

### Data analysis

The 34 lists of NEAST of ecoregions were used to evaluate geographical distributions of angiosperms and to analyze floristic similarities among Taiwan and ecoregions. The NEASTs were transformed into presence data in ecoregions (see Additional file 1: Appendix S1). The presence data of NEASTs were used for the analysis in this study to minimize the bias of data information (Beck et al. 2014; Yesson et al. 2007). NEASTs were evaluated their geographical distribution patterns in Asia. Geographical distribution range of a NEAST was represented by the collection of ecoregions that a NEAST is present. The ecoregions in Asia were categorized into three groups, tropical, subtropical and temperate, according to the published description of ecoregions in WWF. The tropical, subtropical and temperate ecoregions were further subdivided into several sub-regions in this study. The tropical ecoregions were divided into two sub-regions that were ecoregions at tropical continents and tropical islands. The subtropical ecoregions were divided into four sub-regions that were southern slopes of Himalaya, southwest China, southeast China, and southern islands of Japan. The temperate ecoregions were divided into Qinghai-Tibet Plateau and Takla-Makan-Gobi Desert, Mongolia and Siberia, northeast China, Korea peninsula, and northern islands of Japan. Whether the distribution ranges of NEASTs over these sub-regions or not are the division criterions of distribution types.

Floristic similarity between islands and source areas were measured in this study because floristic similarity provides evidences of dispersal routes from source areas to islands (Alsos et al. 2015). In order to analyze the floristic similarities between GLTs and ERGs, the equation c/(c + b) was used to calculate the proportion of species which given GLT shares with ERG. Within the equation, c is the number of shared species and b is the number of species only in given GLT and not in ERG. This measure considers the situation that particular ERG may potentially contain other species which are not in given GLT and which does not necessarily to influence the similarity of that GLT and ERG. The angiosperm lists of 22 GLTs and 34 ecoregions of Asia were combined to make a data matrix for the cluster analysis. Simple nearest neighboring algorithm and Bray–Curtis method were chosen for clustering. The computations were conducted by means of PC-ORD program (v.5) (McCune and Mefford 2006).

## Results

### Numbers of NEAST in ecoregions of Asia

The numbers of family, genus and species of NEAST in ecoregions are showed in Table 2. Four subtropical ecoregions, No. 8, 14, 17 and 20, and one tropical ecoregions, No. 26, possess high numbers of NEAST (Table 2). Three of the four subtropical ecoregions, No. 14, 17, and 20, are the ecoregions at southern China, while the other one at Japan. The tropical ecoregion with high number of NEAST is at Indochina. Subtropical ecoregions at southern China has evidently high floristic relationships with Taiwan. Temperate ecoregions possess relatively lower numbers of NEAST than tropical and subtropical ecoregions demonstrated lower floristic relationships. On the other hand, Philippine is the closest tropical island to Taiwan in distance, but there are only 463 NEASTs in Philippine. The Papua New Guinea is a tropical island distant from Taiwan, while the number of NEAST of Papua New Guinea is higher than that of Philippine. In contrast to tropical islands, angiosperm species richness of subtropical and tropical ecoregions at Asian continent are more closely related to Taiwan.

**Table 2:** The numbers of family, genus, and species of ecoregions in Asia listed in the table were the numbers of non-endemic angiosperm species in Taiwan (NEAST)

Acronym of ecoregions	Family	Genus	Species
Cold winter desert			
ERG-15	65	168	222
ERG-16	28	93	117
Temperate regions			
ERG-02	4	13	14
ERG-03	14	37	43
ERG-01	16	30	33
ERG-04	37	94	115
ERG-05	55	133	170
ERG-06	29	39	46
ERG-07	101	337	495
ERG-11	54	155	190
ERG-13	102	315	456
Subtropical regions			
ERG-08	140	575	1003
ERG-09	120	439	699
ERG-10	82	173	226
ERG-12	126	488	791
ERG-14	155	662	1198
ERG-17	161	841	1631
ERG-20	149	742	1344
ERG-21	104	462	734
Tropical regions			
ERG-18	136	547	886
ERG-19	109	381	551
ERG-23	33	76	96
ERG-25	33	90	112
ERG-27	131	546	917
ERG-28	96	292	423
ERG-22	89	315	486
ERG-24	37	85	108
ERG-26	140	600	1007
ERG-29	104	377	541
ERG-30	100	332	469
ERG-31	75	195	256
ERG-32	68	202	274
ERG-33	96	314	435
ERG-34	121	496	827

The numbers after ERG and dash are the numbers of ecoregions listed in Table 1 and Fig. 2

### Geographical distribution types of NEAST in Asia

**Fig. 3:**
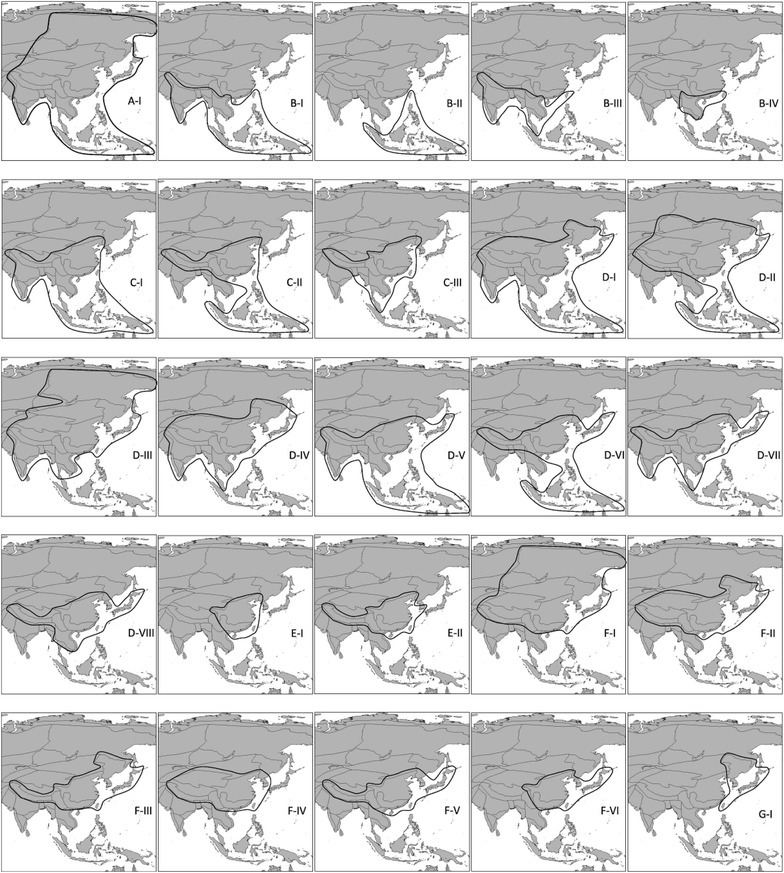
The maps show the 25 distribution types of 7 distribution Groups of non-endemic angiosperm species in Taiwan (NEAST). Angiosperms of Group A distribute from tropical islands to subarctic Siberia. B-I to B-IV are tropical distribution. C-I to C-III are tropical to subtropical distribution. D-I to D-VIII are tropical to temperate distribution. E-I to E-II are subtropical distribution. F-I to F-VI are subtropical to temperate distribution. G is temperate distribution. There are 2428 NEASTs evaluated in this research. The detail descriptions of distribution ranges and the numbers of species are listed in Table 3

Geographical distribution ranges of 3275 angiosperm species were examined in terms of the current occurrences of angiosperm data records (see Additional file 1: Appendix S1). Of the 3275 angiosperms, 847 species are endemic in Taiwan and 2428 are NEASTs. The geographical distribution ranges of the 2428 NEASTs were categorized into 25 types belong to 7 groups (Fig. 3; Table 3).

**Table 3:** Geographical distribution groups of non-endemic angiosperm species in Taiwan (NEAST)

Distribution group	Type	Distribution range	Number of species
Widely distribution (Group A)	A-I	Widely distribution from tropical through subtropical to temperate ecoregions or to cold winter deserts	27
Tropical distribution (Group B)	B-I	Tropical islands, Indochina, India, and few species reach Japan	156
	B-II	Tropical islands	138
	B-III	India and Indochina	27
	B-IV	Indochina, Hainan, southeast coast of China	24
Tropical to subtropical distribution (Group C)	C-I	Tropical islands, Indochina, India, southern slope of Qin-zang Plateau; south, central and north China	398
	C-II	Tropical islands, southern slope of Qing-zang Plateau and south, central and north China	33
	C-III	Indochina, southern slope of Qing-zang Plateau, south, central and north China	232
Tropical to temperate distribution (Group D)	D-I	Tropical islands, Indochina, India, southern slope of Qing-zang Plateau; south, central and north China, Japan, Korea, northeast China	74
	D-II	Tropical islands, Indochina, India, southern slope of Qing-zang Plateau; south, central and north China, Japan, Korea, northeast China, Mongolia, Altai	25
	D-III	From Indochina and India toward north to Siberia	17
	D-IV	Indochina, India, southern slope of Qing-zang Plateau, Xinjiang, south, central, north and northeast China, Japan, Korea	57
	D-V	Tropical islands, Indochina, India, southern slope of Qing-zang Plateau, south, central and north China, Korea and Japan	233
	D-VI	Tropical islands, southern slope of Qing-zang Plateau, south, central and north China, Korea and Japan	46
	D-VII	Indochina, India, southern slope of Qing-zang Plateau, south, central and north China, Korea and Japan	84
	D-VIII	Indochina, southern slope of Qing-zang Plateau, south, central and north China, Korea and Japan	55
Subtropical distribution (Group E)	E-I	Hainan, southeast coast of China, southeast, central and north China	101
	E-II	Hainan, southeast coast of China, southern slope of Qing-zang Plateau, south, central and north China, Korea	164
Subtropical to temperate distribution (Group F)	F-I	Southern slopes of Qing-zang Plateau, Tibet, Xinjiang, south, central, north, and northeast China, Japan, Korea and Siberia	26
	F-II	Hainan, southeast coast of China, Southern slopes of Qing-zang Plateau, Tibet, Xinjiang, south, central, north, and northeast China, Japan, Korea and Siberia	50
	F-III	Southern slopes of Qing-zang Plateau, south, central, and north China Japan and Korea	35
	F-IV	Hainan, southeast coast of China, Southern slopes of Qing-zang Plateau, Tibet, Xinjiang, south, central, north, and northeast China, and Korea	30
	F-V	southeast coast of China, Southern slopes of Qing-zang Plateau, south, central, and north China Japan and Korea	152
	F-VI	Hainan, southeast coast of China, south, central, and north China, Japan	94
Temperate distribution (Group G)	G-I	Northeast China, Japan and Korea, few species reach Hainan, southeast coast of China and tropical islands	150

The NEASTs were categorized into 7 distribution groups and 25 types according to their distribution ranges in Asia. Descriptions of geographical distribution ranges and numbers of angiosperm species of each type were listed in the table

NEASTs of Group B-II has their distribution ranges from tropical islands to Taiwan and these plants are absent at Asian continent. Meanwhile, Group G has their distribution ranges only from temperate ecoregions to Taiwan. Distribution ranges of Group B-II and Group G are absent at tropical and subtropical ecoregions at Asian continent and are obviously different from most of the NEASTs. In contrast to these two groups, more than 88% of all the NEASTs have their distribution ranges over tropical, subtropical or temperate ecoregions at Asian continent.

### Cluster analysis and floristic similarities among GLTs and ecoregions

Floristic similarities of pairs between 22 GLTs and 34 ecoregions in Asia are listed in Table 4. Floristic similarities are evidently low between all the GLTs and ecoregions at higher latitudes. The values of similarity indices between all the GLTs and ecoregions at higher latitudes, from ERG-01 to ERG-06, are less than 0.1. Higher floristic similarities are observed between some GLTs and 5 subtropical and 2 tropical ecoregions, they are No. 08, 14, 17, 18, and 20. The second higher floristic similarities are observed between some GLTs, especially the GLTs at lower elevations, and tropical ecoregions, No. 26, 27, and 34. Interestingly, similarities indices between GLTs at higher elevations and subtropical ecoregions are higher than that between GLTs at higher elevations and temperate ecoregions.

**Table 4:** Floristic similarities between geographical localities of Taiwan (GLT) and 34 terrestrial ecoregions (ERG) in Asia

Number of ecoregion	Cold winter desert		Group of temperate ecoregions									Group of subtropical ecoregions							
	ERG-15	ERG-16	ERG-01	ERG-02	ERG-03	ERG-04	ERG-05	ERG-06	ERG-07	ERG-11	ERG-13	ERG-08	ERG-09	ERG-10	ERG-12	ERG-14	ERG-17	ERG-20	ERG-21
1. NE-500	0.08	0.05	0.02	0.00	0.02	0.05	0.04	0.02	0.18	0.09	0.16	*0.36*	0.27	0.08	0.28	*0.49*	*0.65*	*0.50*	0.29
2. NW-500	0.08	0.04	0.01	0.00	0.02	0.05	0.06	0.02	0.19	0.08	0.19	*0.41*	0.31	0.10	0.32	*0.51*	*0.65*	*0.52*	0.29
3. ME-500	0.08	0.03	0.01	0.00	0.01	0.04	0.04	0.01	0.15	0.06	0.17	*0.36*	0.27	0.09	0.26	*0.49*	*0.65*	*0.52*	0.29
4. MW-500	0.07	0.04	0.01	0.00	0.02	0.04	0.03	0.01	0.15	0.07	0.18	*0.36*	0.26	0.08	0.27	*0.45*	*0.63*	*0.53*	0.31
5. SE-500	0.07	0.03	0.01	0.00	0.01	0.03	0.03	0.01	0.12	0.05	0.15	0.30	0.22	0.07	0.22	*0.42*	*0.60*	*0.51*	0.28
6. SW-500	0.07	0.04	0.01	0.00	0.01	0.04	0.04	0.01	0.14	0.07	0.16	0.34	0.24	0.08	0.26	*0.43*	*0.63*	*0.54*	0.31
7. ST-500	0.05	0.03	0.01	0.00	0.01	0.02	0.03	0.01	0.11	0.05	0.12	0.29	0.20	0.08	0.21	*0.36*	*0.54*	*0.44*	0.25
8. NE-1500	0.07	0.03	0.01	0.00	0.01	0.04	0.06	0.02	0.18	0.06	0.16	*0.36*	0.27	0.09	0.28	*0.47*	*0.59*	*0.47*	0.23
9. NW-1500	0.08	0.03	0.01	0.00	0.01	0.04	0.05	0.01	0.18	0.06	0.18	*0.36*	0.28	0.11	0.26	*0.51*	*0.63*	*0.51*	0.24
10. ME-1500	0.07	0.03	0.01	0.00	0.01	0.04	0.05	0.01	0.16	0.06	0.18	0.34	0.26	0.10	0.25	*0.48*	*0.58*	*0.49*	0.23
11. MW-1500	0.07	0.04	0.01	0.00	0.01	0.04	0.05	0.02	0.18	0.07	0.17	*0.36*	0.26	0.09	0.27	*0.46*	*0.61*	*0.50*	0.26
12. SE-1500	0.06	0.03	0.00	0.00	0.00	0.03	0.03	0.01	0.13	0.05	0.14	0.31	0.23	0.09	0.23	*0.45*	*0.60*	*0.50*	0.23
13. SW-1500	0.06	0.02	0.01	0.00	0.01	0.03	0.03	0.01	0.12	0.05	0.14	0.29	0.20	0.08	0.20	*0.42*	*0.58*	*0.47*	0.23
14. ST-1000	0.04	0.01	0.00	0.00	0.00	0.01	0.00	0.00	0.05	0.03	0.06	0.21	0.14	0.08	0.12	0.32	*0.50*	*0.39*	0.16
15. NE-2500	0.10	0.05	0.01	0.01	0.02	0.05	0.08	0.03	0.22	0.07	0.18	*0.37*	0.28	0.10	0.29	*0.49*	*0.55*	*0.45*	0.22
16. NW-2500	0.10	0.05	0.01	0.00	0.02	0.06	0.08	0.03	0.24	0.07	0.20	*0.40*	0.32	0.11	0.31	*0.52*	*0.58*	*0.47*	0.23
17. ME-2500	0.09	0.04	0.01	0.01	0.01	0.05	0.07	0.02	0.20	0.06	0.20	*0.36*	0.28	0.10	0.27	*0.50*	*0.56*	*0.47*	0.22
18. MW-2500	0.09	0.05	0.01	0.01	0.02	0.05	0.08	0.02	0.21	0.07	0.18	*0.35*	0.27	0.09	0.28	*0.49*	*0.58*	*0.48*	0.24
19. S-2500	0.07	0.02	0.01	0.00	0.01	0.03	0.04	0.02	0.15	0.04	0.13	0.29	0.22	0.09	0.21	*0.43*	*0.55*	*0.43*	0.20
20. N-3500	0.10	0.06	0.01	0.01	0.02	0.06	0.09	0.03	0.22	0.08	0.19	0.34	0.25	0.08	0.28	*0.46*	*0.49*	*0.42*	0.21
21. M-3500	0.10	0.05	0.01	0.01	0.02	0.05	0.09	0.03	0.21	0.08	0.19	0.34	0.26	0.10	0.28	*0.45*	*0.49*	*0.42*	0.21
22. S-3500	0.04	0.03	0.00	0.00	0.01	0.03	0.07	0.01	0.15	0.04	0.12	0.26	0.21	0.07	0.19	0.34	*0.37*	0.33	0.15

Number of ecoregion	Group of tropical ecoregions														
	ERG-18	ERG-19	ERG-22	ERG-23	ERG-24	ERG-25	ERG-26	ERG-27	ERG-28	ERG-29	ERG-30	ERG-31	ERG-32	ERG-33	ERG-34
1. NE-500	*0.38*	0.21	0.17	0.04	0.07	0.03	*0.40*	0.33	0.13	0.15	0.17	0.06	0.09	0.14	0.27
2. NW-500	*0.40*	0.24	0.20	0.04	0.05	0.05	*0.41*	*0.36*	0.17	0.20	0.18	0.09	0.11	0.17	0.33
3. ME-500	*0.41*	0.27	0.20	0.04	0.05	0.05	*0.43*	*0.37*	0.16	0.22	0.21	0.12	0.12	0.19	0.34
4. MW-500	*0.44*	0.29	0.24	0.05	0.06	0.06	*0.45*	*0.42*	0.19	0.23	0.22	0.11	0.13	0.20	*0.38*
5. SE-500	*0.43*	0.30	0.21	0.04	0.06	0.05	*0.44*	*0.41*	0.20	0.25	0.23	0.13	0.14	0.21	*0.36*
6. SW-500	*0.45*	0.29	0.26	0.06	0.06	0.06	*0.47*	*0.45*	0.22	0.25	0.23	0.11	0.14	0.22	*0.41*
7. ST-500	*0.38*	0.27	0.21	0.05	0.05	0.05	*0.41*	*0.39*	0.19	0.26	0.23	0.12	0.13	0.21	*0.37*
8. NE-1500	0.33	0.21	0.13	0.03	0.04	0.03	0.35	0.29	0.12	0.16	0.15	0.08	0.08	0.13	0.24
9. NW-1500	*0.35*	0.23	0.13	0.03	0.04	0.04	*0.37*	0.30	0.11	0.16	0.15	0.08	0.08	0.13	0.21
10. ME-1500	0.31	0.19	0.12	0.02	0.04	0.03	0.34	0.27	0.11	0.16	0.14	0.08	0.08	0.13	0.21
11. MW-1500	0.35	0.21	0.17	0.03	0.05	0.04	*0.38*	0.31	0.14	0.18	0.17	0.09	0.09	0.16	0.27
12. SE-1500	*0.36*	0.24	0.14	0.03	0.04	0.04	*0.39*	0.33	0.14	0.19	0.16	0.11	0.10	0.16	0.24
13. SW-1500	*0.36*	0.24	0.15	0.03	0.05	0.04	*0.39*	0.34	0.14	0.19	0.17	0.10	0.10	0.15	0.25
14. ST-1000	*0.37*	0.29	0.15	0.03	0.04	0.03	*0.35*	0.34	0.16	0.20	0.17	0.11	0.11	0.16	0.22
15. NE-2500	0.24	0.14	0.09	0.02	0.03	0.02	0.25	0.18	0.07	0.11	0.09	0.06	0.05	0.07	0.15
16. NW-2500	0.25	0.14	0.09	0.02	0.03	0.02	0.26	0.19	0.07	0.11	0.09	0.06	0.05	0.08	0.16
17. ME-2500	0.24	0.14	0.11	0.03	0.04	0.03	0.27	0.18	0.06	0.13	0.09	0.06	0.04	0.08	0.15
18. MW-2500	0.29	0.17	0.12	0.02	0.03	0.03	0.30	0.23	0.09	0.13	0.12	0.07	0.07	0.10	0.19
19. S-2500	0.30	0.19	0.11	0.02	0.04	0.03	0.31	0.26	0.10	0.16	0.12	0.08	0.07	0.11	0.17
20. N-3500	0.17	0.08	0.07	0.01	0.01	0.02	0.18	0.12	0.04	0.09	0.05	0.04	0.04	0.04	0.12
21. M-3500	0.19	0.12	0.08	0.02	0.03	0.02	0.21	0.15	0.05	0.11	0.07	0.05	0.05	0.06	0.15
22. S-3500	0.13	0.08	0.05	0.00	0.01	0.02	0.20	0.13	0.03	0.07	0.04	0.04	0.02	0.02	0.07

The index was calculated by equation c/(c + b), c is the species shares between given GLT and ERG and b is the species only in given GLT. The columns are the ecoregions as in Fig. 2. The row names are the abbreviation of GLTs as in Fig. 1. The italics in the table are the values of similarity indices higher than 0.35

**Fig. 4:**
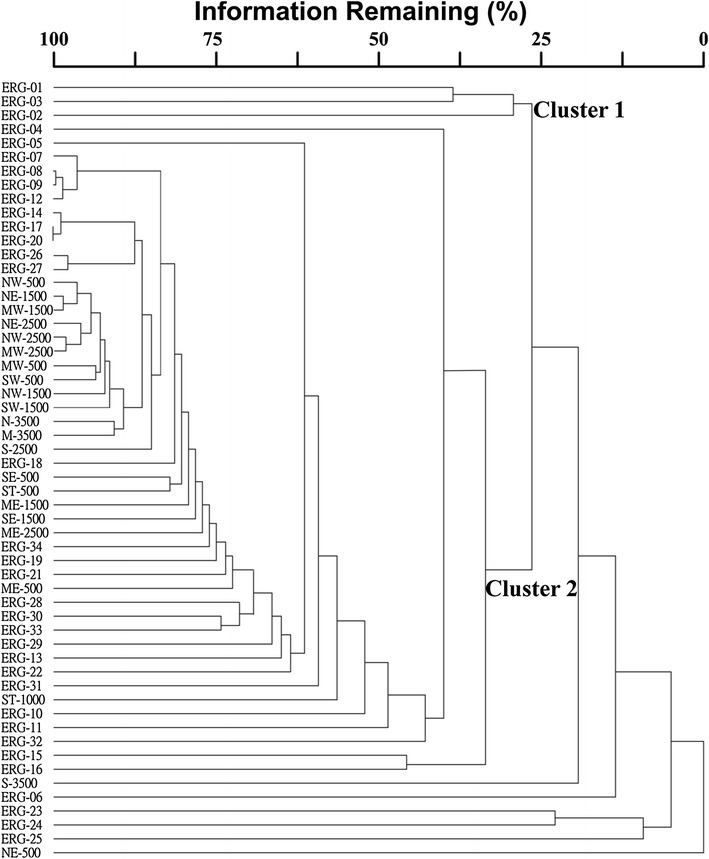
Floristic relationships between 22 geographical localities of Taiwan (GLT) and 34 ecoregions in Asia were analyzed by cluster analysis, using simple nearest neighbor algorithm and Bray–Curtis dissimilarity method. Non-endemic angiosperm species in Taiwan (NEAST) were used for the cluster analysis. The ecoregions of cold winter desert and temperate needle-leaved forests were the first cluster and came out as the most deviating regions. The second cluster was comprised of many ecoregions and GLTs. Deep in the second cluster, tropical and subtropical ecoregions and some GLTs were grouped together. ERG is the acronym of ecoregion. The *numbers* after ERG and *dash* are the number of ecoregions listed in Table 1 and Fig. 2. The number and acronyms of 22 GLTs are the same as in Fig. 1

Floristic similarities among GLTs and subtropical and tropical ecoregions in Asia are supported by the cluster analysis. Simple nearest neighboring algorithm resulted 2 major clusters (Fig. 4). Temperate and cold-winter desert ecoregions at Asian continent were came out as the first cluster and were the most deviating ecoregions. Many tropical, subtropical ecoregions and GLTs formed the second cluster. Deep in the second cluster, three subtropical ecoregions, two tropical ecoregions, and many GLTs were grouped together.

The GLTs at northern Taiwan and high elevations were assumed to have floristic similarities with temperate ecoregions. However, the assumption is not supported by our analysis. The results of floristic similarity and clustering analysis both concluded floristic dissimilarity among GLTs at higher elevations and ecoregions at higher latitudes in Asia continent. Although some angiosperms in GLTs at higher elevation are observed in higher latitudes, they are not dominant members. A further examination on the floristic lists of GLTs demonstrated that angiosperms with tropical and subtropical distributions (Group B, C, D, E, F) extend their distribution ranges from low to high elevations in Taiwan (see later texts). This is most likely the explanation of the low floristic similarity between Taiwan and temperate ecoregions in Asian continent.

### Proportions of geographical distribution types of NEAST in Taiwan

**Fig. 5:**
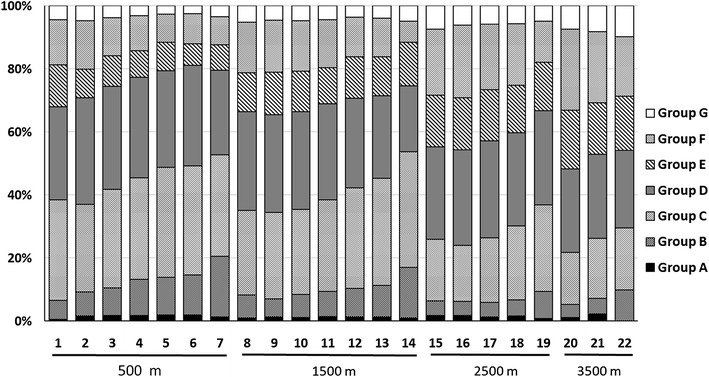
The proportions of angiosperms with seven geographical distribution groups show different trends among geographical localities of Taiwan (GLTs). Angiosperms with tropical and subtropical distribution are dominant members in all the GLTs. The proportions of angiosperms with tropical distributions (Group B, C and D) increase from northern to southern Taiwan but decrease from low to high elevations. The proportions of angiosperms with subtropical distributions (Group E and F) present opposite trends to that with tropical distributions. Angiosperms with temperate distribution (Group G) are not dominant members of GLTs at higher elevations. The numbers at *X axis* are the numbers of GLTs listed in Fig. 1. At *bottom of the figure*, four lines and four numbers *below the lines* represent the upper altitudinal limits of GLTs

NEASTs with tropical and subtropical geographical distribution ranges (Group B, C, D, E, and F) are the dominant members of all the GLTs (Fig. 5). The 7 geographical distribution groups exhibit similar ratio among GLTs, though slightly differences has been observed. The proportions of Group B, C and D are relatively higher in southern GLTs at S and ST latitude zones, while that of Group E and F in northern GLTs at N latitude zone and higher elevations. The southern GLTs at S and ST latitude zones possess relatively more NEASTs with tropical distributions, whereas the northern GLTs at N latitude zone more NEASTs with subtropical distributions.

The GLTs above 3000 m ASL were expected to be dominated by NEASTs with temperate distributions (Group G); however, they are dominated by NEASTs with tropical and subtropical geographical distribution ranges. In other words, NEASTs with tropical and subtropical distributions extend their ranges from low to high elevations in Taiwan that have caused low floristic similarities among GLTs at high elevations and ecoregions at higher latitudes (Fig. 4; Table 4). Results from geographical distribution range, cluster analysis and similarity indices all concluded floristic similarities among GLTs and tropical and subtropical ecoregions in Asian continent.

## Discussion

Recently, studies on large scale biodiversity pattern are available because of rapid accumulation of global data records (Beck et al. 2014; Flemons et al. 2007; Guralnick et al. 2007; Ingwersen and Chavan 2011; Saarenmaa 2005). GBIF is the largest online provider of global data records (Flemons et al. 2007; Padial et al. 2010). Large quantities of biodiversity data are inevitably spatially biased due to uneven effort of data collections and it is problematic to use species occurrence data in species distribution models (Beck et al. 2014). Subsampling distribution data had been used to reduce spatial bias of data and improved model quality (Beck et al. 2014). Our study attempts to minimize the bias error of species occurrence data by transforming georeferenced data into presence-absence data. Our results of floristic relationships between GLTs and ecoregions in Asia are acceptable because of few reasons. The first is that the numbers of scientific names are higher at subtropical ecoregions in this study. The species richness pattern presented by the numbers of scientific names in Table 1 agreed with the proposed latitudinal pattern of species richness in Asia. Subtropical region in East Asia were proposed to have high species richness along the latitudinal gradient (Feng et al. 2016; López-Pujol et al. 2011; Qiu et al. 2011; Zhu 1997). In this study, higher numbers of scientific names in two subtropical ecoregions agreed with the proposed latitudinal change of species richness, despite the numbers of data records are not the highest (Table 1). In addition, not all the angiosperm species of ecoregions were utilized in this study but only the species that are common between Taiwan and ecoregions, the NEASTs. The utilization of species common between Taiwan and ecoregions diminished the effects of spatial bias of data collections and minimized the study error caused by spatial bias of database.

Interestingly, our study has implied an extraordinary species richness pattern along latitudes in East Asia. A latitudinal pattern of angiosperm species richness appears while numbers of scientific names are compared among ecoregions of four groups (Table 1). Two subtropical ecoregions in South China possess the highest number of scientific names in Asia and there are 12,271 and 11,696 scientific names of ecoregions 20 and 17, respectively (Table 1). High numbers of scientific names in subtropical ecoregions are expected to have high angiosperm species richness.

Generally, species richness is highest at tropical regions and decreases toward higher latitudes (Barthlott et al. 2007; Pianka 1966; Qian et al. 2003, 2007). To our knowledge, higher angiosperm species richness in subtropical regions than in tropical regions is a novel species richness pattern along latitudes. Higher species richness at subtropical region observed in this study might be false because some factors may lead to a false species richness pattern along latitudes. The factors include bias data collections, synonyms in lists of ecoregions, various sizes of ecoregions’ areas, etc. Although these factors do not support our observation, our observation cannot be rejected because of some reasons. The first, subtropical region had been proposed as one of the biodiversity centers in Asia since several decades ago (Wang 1992a, b). The subtropical region had served as a refuge for angiosperms in Asia during ice age in the Quarternary (Qian and Ricklefs 2000; Qian et al. 2003; Qiu et al. 2011; Tiffney 1985). These studies had offered evidences supporting high species richness of subtropical regions in Asia. The second, a recent study had also implied higher plant species richness at subtropical regions. The study proposed that species richness of family Fagaceae is higher at subtropical regions than at tropical or temperate regions in East Asia (Liao and Chen 2015). The study of Fagaceae species richness is similar to our results in Table 1. In summary, angiosperm species richness along latitudes in Asia is worth to be investigated to understand the patterns and causal factors of species richness along latitudes in Asia.

Latitudinal patterns of angiosperm species richness in Asia are important on the angiosperm specie richness in Taiwan because latitudinal range and climatic environments of Taiwan are similar to the subtropical regions in continental Asia. However, subtropical region is likely not the only source area of insular non-endemic angiosperm species in Taiwan. Some phylogenetic studies investigated population genetic variations to evaluate historical migration processes of plants from neighboring regions to Taiwan and five hypothetical migration routes have been proposed (Fig. 2) (Huang 2011, 2014; Shen 1997; Wang 1992a, b). The five routes provided pathways for plants to migrate from tropical islands (Route I), Indochina (Route II), southern China (Route III), northeast China (Route IV) and temperate islands (Route V) to Taiwan (Fig. 2). Our study attempts to identify the extent to which the five migration routes affected on the angiosperm species richness of Taiwan.

Our results of floristic similarities and geographical distributions both demonstrated that angiosperm species richness of Taiwan are closely related to tropical ecoregions at Indochina and subtropical ecoregions at southern China. The results support that the Route II and Route III are important on the angiosperm species richness in Taiwan. The Route II provided pathway for the migration of plants from tropical ecoregions or Indochina through Southern China Sea to Taiwan (Huang 2014; Shen 1997) and angiosperms of Group B, C, and D was able to migrate to Taiwan through Route II. The Route III is from the eastern slope of Qinghai-Tibet Plateau through southern China to Taiwan (Matuszak et al. 2016; Wang 1992a, b). The Route III provided the pathway for the migration of plants from subtropical ecoregions to Taiwan and angiosperms of Group C, D, E, and F were able to migrate to Taiwan through Route III.

The plants of tropical islands had presumably migrated to Taiwan via long distance dispersal and angiosperms of B-II probably migrated to Taiwan by using Route I. However, tropical islands in south Asia, include Philippine and New Guinea, are not as important as Asian continents on the angiosperm species richness of Taiwan because of two reasons. The first, the number of NEAST is lower in tropical islands than in Asian continent. The second, floristic relationships between Taiwan and tropical islands are not as close as that between Taiwan and Asian continents. Land bridge connections had never existed between Philippine and Taiwan in the Quaternary (Voris 2000; Zeng 1993); therefore, plants migrated from tropical islands to Taiwan were probably dispersed by currents, winds or birds. It is likely because of that migration of plants through long distance dispersal is more difficult than that through land bridge connections.

In contrast to tropical and subtropical ecoregions, temperate ecoregions are far less important on angiosperm species richness in Taiwan. The Route IV and Route V provided pathways for the migration of plants from temperate ecoregions to Taiwan (Fig. 2). The Route IV is from northeast China through Korea, Yellow Sea, East China Sea to Taiwan (Shen 1997). The Route V is from Japan through Ryukyu archipelago to Taiwan (Huang and Lin 2006). The angiosperms of Group D and F extend distribution ranges from tropical or subtropical to temperate ecoregions had probably migrated to Taiwan through Route IV or V. However, it is questionable whether angiosperms of Group D and F had migrated from southern to northern latitudes or vice versa. A published paper had proposed several hypothetical migration routes from southern to northern latitudes in China (Wang 1992a, b). Angiosperms had migrated from Southwest China toward north, northeast, or east to northern China, northeast China, Japan or Taiwan, respectively (Wang 1992a, b). Therefore, angiosperms with distribution from tropical or subtropical to temperate ecoregions (Group D and F) had probably migrated from southern to northern latitudes or from southern China to Taiwan. The studies of Wang (1992a, b) had implied that most of the plants with distributions from tropical or subtropical to temperate regions had probably migrated from southern to northern latitudes. Therefore, Route IV and V were not supported by the studies of Wang (1992a, b). Although few evidences had indicated migrations of gymnosperms from temperate regions to southern latitudes (Li et al. 2003), more evidences are necessary for the identification of Route IV and Route V for the migration of angiosperms.

Angiosperms of Group G have not been observed at tropical or subtropical ecoregions in Asian continent. These plants had probably migrated from northern latitudes to Taiwan or vice versa. Migration of plants from northern latitudes to Taiwan was supported by the phylogenetic studies of *Chamaecyparis formosensis* and *Chamaecyparis obtusa* var. *formosana* (Li et al. 2003). Migration of plants from northern to southern latitudes might have been caused by global cooling in the late Tertiary and Quaternary that had forced many plants to migrate southward (Chung et al. 2011; Denk 2004; Huang and Lin 2006; Huang et al. 2004). Meanwhile, phylogenetic study had identified that *Trochodendron aralioides* had expanded from Taiwan to northern latitudes after ice age (Huang and Lin 2006). Migrations of plants from Taiwan to northern latitudes or vice versa were supported by some evidences of published documents. Analysis on the population genetics is suggested to provide evidence for identifying the migration of plants from northern latitudes to Taiwan or vice versa; to date, few study has focused on this topic. Route IV and V had provided pathway for plants to migrate from temperate regions to Taiwan, whereas there are only 150 species of Group G. The number of Group G indicated that Route IV and V are less important on angiosperm species richness in Taiwan.

Our results concluded that tropical and subtropical regions at Asian continent were most important on the angiosperm species richness in Taiwan because of high floristic similarity between GLTs and tropical and subtropical ecoregions. Tropical and subtropical ecoregions are most likely the most important source areas of angiosperms in Taiwan. The tropical islands at South Asia are the second important. Angiosperms with distribution ranges over tropical and subtropical ecoregions are the dominant members of the vegetation from low to high elevations in Taiwan. Most of the angiosperms at high elevation in Taiwan are the species with tropical and subtropical distributions and, therefore, temperate ecoregions in Asia are less important on the angiosperm species richness in Taiwan. Infraspecific genetic variations of angiosperms in Asia is suggested to be investigated to determine the historical migration of angiosperms from tropical or subtropical regions to Taiwan.

## Additional file

**Additional file 1: Appendix S1.** Presence-absence data of angiopserms examined in this study.

## Abbreviations

ASL: above sea level
GBIF: Global Biodiversity Information Fertility
GLT: geographical locality of Taiwan
NEAST: non-endemic angiosperm species in Taiwan
WWF: World Wild Fund
